# The association between irregular working hours and cardiovascular diseases in a multi-ethnic population: the HELIUS study

**DOI:** 10.1093/occmed/kqaf069

**Published:** 2025-07-11

**Authors:** Gilbert W M Wijntjens, Ali Dehghani, Ehsan Motazedi, Sophia Franklin, Jaap J J Maas, Henrike Galenkamp, Bert-Jan H van den Born, Frederieke G Schaafsma

**Affiliations:** Department of Public and Occupational Health, Amsterdam Public Health Research Institute, Amsterdam UMC, Amsterdam, 1100 DD, The Netherlands; Department of Public and Occupational Health, Amsterdam Public Health Research Institute, Amsterdam UMC, Amsterdam, 1100 DD, The Netherlands; Department of Public and Occupational Health, Amsterdam Public Health Research Institute, Amsterdam UMC, Amsterdam, 1100 DD, The Netherlands; Department of Public and Occupational Health, Amsterdam Public Health Research Institute, Amsterdam UMC, Amsterdam, 1100 DD, The Netherlands; Department of Public and Occupational Health, Amsterdam Public Health Research Institute, Amsterdam UMC, Amsterdam, 1100 DD, The Netherlands; Department of Public and Occupational Health, Amsterdam Public Health Research Institute, Amsterdam UMC, Amsterdam, 1100 DD, The Netherlands; Department of Public and Occupational Health, Amsterdam Public Health Research Institute, Amsterdam UMC, Amsterdam, 1100 DD, The Netherlands; Department of Vascular Medicine, Amsterdam Cardiovascular Sciences, Amsterdam UMC, Amsterdam, 1105 AZ, The Netherlands; Department of Public and Occupational Health, Amsterdam Public Health Research Institute, Amsterdam UMC, Amsterdam, 1100 DD, The Netherlands

## Abstract

**Background:**

Irregular working hours are a risk factor for cardiovascular diseases (CVD).

**Aims:**

We studied ethnic disparities in the association between irregular working hours and CVD, as well as the mediating stress-related pathways.

**Methods:**

Logistic regression was used to study the cross-sectional association between (a history of) irregular working hours and prevalent CVD (self-reported myocardial infarction, dotter/bypass operation or stroke) in 18 284 participants (18–71 years) in a population of Dutch, South-Asian Surinamese, African Surinamese, Ghanaian, Turkish and Moroccan origin from the HELIUS study. We considered three mediator models adjusting for behavioural, physiological and psychosocial stress. We tested for interaction between irregular working hours and ethnicity and stratified the analysis by ethnicity.

**Results:**

The prevalence of CVD was 18% (717 out of 4041) and 14% (1963 out of 14 243) in individuals with versus without irregular working hours. Working irregular hours was associated with prevalent CVD (OR 1.27, 95% CI 1.15–1.40) after adjusting for sociodemographic confounders. There was a significant interaction between ethnicity and irregular working hours on CVD. Strongest associations with prevalent CVD were found for South-Asian Surinamese (OR 1.47, 95% CI 1.18–1.82), African Surinamese (OR 1.29, 95% CI 1.06–1.57) and Moroccans (OR 1.43, 95% CI 1.11–1.84). There were considerable ethnic differences in the mediating stress-related pathways that associate irregular working hours with prevalent CVD.

**Conclusions:**

Working irregular hours is associated with an increased prevalence of CVD in a multi-ethnic population. We show ethnic disparities in the associations between irregular working hours and CVD, as well as in the stress-related pathways that mediate these associations.

Key learning points **What is already known about this subject:**

- Individuals with irregular working hours have an increased risk of cardiovascular diseases.
- There are considerable ethnic disparities in cardiovascular and circadian health.

**What this study adds:**

- This study shows that irregular working hours are associated with increased prevalence of cardiovascular diseases in a multi-ethnic population.
- We show ethnic disparities in the association between irregular working hours and cardiovascular diseases, as well as in the stress-related pathways that mediate this association.

**What impact this may have on practice or policy:**

- Ethnicity-tailored preventive strategies may have the potential to improve the prevention of cardiovascular diseases in individuals with irregular working hours.
- Future studies should explore the potential of ethnicity-­tailored preventive strategies.

## INTRODUCTION

Cardiovascular diseases (CVD) are currently the leading cause of morbidity and mortality worldwide [1, 2]. Although the global age-standardized CVD mortality rates have declined steadily since 1990 due to the implementation of preventive strategies, the decline has recently plateaued in high-income countries [2, 3]. Moreover, risk prediction models based on the traditional CVD risk factors are not optimally calibrated to the observed incidence curves [4]. Therefore, a critical revision of our preventive strategies, as well as novel scientific insights regarding non-traditional CVD risk factors, is timely needed. In the European Union Labour Force Survey, irregular working hours, or shift work, are defined as work schedules that deviate from standard daytime hours (8 a.m. to 6 p.m.) on weekdays [5]. On average, one-fifth of the European workforce works in shifts [6], and non-standard work schedules are more common among ethnic minorities [7, 8]. There is abundant evidence that individuals with irregular working hours have an increased risk for CVD [9, 10]. However, it remains unclear whether there are ethnic disparities in the relationship between irregular working hours and CVD, or in the stress-related pathways that mediate this link. This unclarity is despite well-documented ethnic disparities in CVD and circadian health [11–14]. Therefore, the present study aims to investigate ethnic disparities in the relation between irregular working hours and CVD risk in a multi-ethnic population living in Amsterdam, the Netherlands.

## METHODS

The present study included baseline data from the HELIUS (Healthy Life in an Urban Setting) study. The aims and design of the HELIUS study have been described in detail previously [15]. In brief, HELIUS is a prospective cohort study including individuals aged 18–70 years from different ethnic groups (Dutch, Surinamese, Ghanaian, Turkish and Moroccan origin) living in Amsterdam, the Netherlands. Participants were randomly sampled from the municipal registers, stratified by ethnicity. The study was approved by the ethical committee of the Amsterdam Medical Center of the University of Amsterdam (NL32251.018.10), and all participants provided written informed consent.

Baseline data collection was performed between 2011 and 2015 and included physical examinations and structured questionnaires on demographic, socioeconomic, and proximal risk factors for CVD. Questionnaire and physical examination data were available in 22 162 participants. We excluded participants with unknown/other ethnic origin (*N* = 48) or Surinamese origin other than African or South-Asian origin (*N* = 500) due to the small sample size. We also excluded participants with no information regarding irregular working hours (*N* = 2902). Hence, the complete study population included 18 746 participants.

Ethnic origin was defined based on the country of birth of individuals and of their parents. In detail, an individual was defined as of non-Dutch ethnic origin if he or she fulfilled one of two criteria: (1) the individual was born outside the Netherlands and has at least one parent born outside the Netherlands (first generation), or (2) the individual was born in the Netherlands but both parents were born outside the Netherlands (second generation) [16]. For the Dutch sample, we invited people who were born in the Netherlands and whose parents were born in the Netherlands. A limitation of the country of birth indicator for ethnicity is that people who are born in the same country might have a different ethnic background, which in the Dutch context is applicable to the Surinamese population. Therefore, after data collection, participants of Surinamese ethnic origin were further classified according to self-reported ethnic origin into ‘African’, ‘South-Asian’, ‘Javanese’ or ‘other’.

Information regarding irregular working hours and prevalent CVD was collected from the HELIUS questionnaires: Do you have to (or had to) work during irregular hours, such as night work? The frequency of irregular working hours was defined as the number of hours per week. Prevalent CVD was defined as a combined clinical endpoint of any self-reported prior myocardial infarction (including >half-hour chest pain), angioplasty or bypass surgery of the coronary arteries or bypass surgery of the lower extremities, or stroke (cerebral infarction or cerebral haemorrhage).

The occupational level was classified as missing, elementary, lower, middle or secondary, higher, or scientific according to the Dutch Standard Occupational Classification system [17]. Alcohol consumption was defined as alcohol intake in the past 12 months (yes/no). Smoking status was categorized as yes, no or ex-smoker. Habitual physical activity was measured using the SQUASH questionnaire and categorized according to the Dutch guideline for physical activity by summing up the number of days per week for each moderate-intensity and high-intensity activity lasting at least 30 min. The SQUASH questionnaire assesses the level of occupational, transport, household, and leisure-time physical activity [18]. A total of ≥5 days resulted in participants being categorized as achieving the Dutch norm for physical activity. Central obesity was defined as a waist circumference ≥80 cm for females, ≥90 cm for South-Asian Surinamese males, and ≥94 cm for non-South-Asian Surinamese males. Short sleep was defined as an average of <7 h of sleep per night. Hypertension was defined as a systolic blood pressure ≥140 mmHg, a diastolic blood pressure ≥90 mmHg, or self-reported use of blood pressure-lowering medication (Anatomical Therapeutic Chemical (ATC) codes C02, C03, C07, C08 and C09). Dyslipidaemia was defined as either self-reported use of lipid-lowering agents (ATC code C10) or cholesterol/HDL-ratio ≥5. Diabetes mellitus was defined as increased fasting glucose (≥7 mmol/l) or self-reported use of blood glucose-lowering medication (ATC code A10). Psychosocial stress was based on two questions regarding experienced stress in the past 12 months, and defined as feeling irritable or anxious or having trouble sleeping because of the situation at work/at home using an ordinal scale to describe the presence of no, some, several or permanent stress at work/home, or ‘not applicable at work’ [19].

We used logistic regression to investigate associations between irregular working hours and prevalent CVD. Model 1 was adjusted for the sociodemographic confounders: age, sex, occupational level and ethnicity (the reference model). We considered three mediator-adjusted models as a proxy for the mediating stress-related pathways that associate irregular working hours with CVD: In Models 2– 4, behavioural stress (physical inactivity, smoking, alcohol consumption and central obesity; Model 2), physiological stress (hypertension, diabetes mellitus, dyslipidaemia and short sleep; Model 3), and psychosocial stress (experienced stress at home/work; Model 4), were separately added to Model 1. In Model 3, we separated short sleep from the other physiological stress factors to compare the mediating effect of short sleep with the other stress factors. Finally, we looked into a model with sociodemographic confounders and all mediators (Model 5). We also studied the association between the frequency of irregular working hours (no versus ≤20 h versus >20 irregular working hours per week) and prevalent CVD. The lower bound of the natural indirect effect (NIE lower bound) of the mediating stress factors was calculated as a measure of mediation using the ‘difference method’: Odds ratio (OR)[model1]/OR[model2–4] [20]. A NIE > 1.000 indicates an indirect effect on the outcome. The logistic regression analysis was first performed in the entire study population, whereafter we assessed the interaction between irregular working hours and ethnicity on CVD. Because of a significant interaction (Dutch versus South-Asian Surinamese, OR 1.41, 95% CI 1.01–1.98; Table 1, available as Supplementary data at *Occupational Medicine* Online), we stratified the logistic regression analysis per ethnic group. *P*-values <0.05 were considered significant. Data were analysed using Stata/SE version 15.1.

**Table 1.** Sociodemographic and clinical characteristics

	Total sample	No irregular working hours	Irregular working hours
*N*	18 284	14 243	4041
Irregular working hours			
Hours per week (Q1, Q3)^a^	–	–	24 (9, 38)
Ethnicity, *N* (%)			
Dutch	4311 (24)	3438 (24)	873 (22)**
South-Asian Surinamese	2704 (15)	2043 (14)	661 (16)**
African Surinamese	3776 (21)	2705 (19)	1071 (27)**
Ghanaian	1925 (11)	1546 (11)	379 (9)**
Turkish	2706 (15)	2212 (16)	494 (12)**
Moroccan	2862 (17)	2299 (16)	563 (14)**
Sociodemographics			
Age, years (SD)	44 (12)	44 (12)	45 (12)**
Female, *N* (%)	9990 (55)	8379 (59)	1611 (40)**
Occupational level, *N* (%)			
Missing	653 (4)	504 (4)	149 (4)
Elementary	2766 (15)	2284 (16)	482 (12)**
Lower	5244 (29)	3772 (27)	1472 (36)**
Medium	4681 (26)	3515 (25)	1166 (29)**
Higher	3593 (20)	3008 (21)	585 (15)**
Scientific	1347 (7)	1160 (8)	187 (5)**
Behavioural stress, *N* (%)			
Physical inactivity	7573 (41)	6119 (43)	1454 (36)**
Smoking			
Never-smoker	9685 (53)	7875 (55)	1810 (45)**
Ex-smoker	3985 (22)	3038 (21)	947 (23)**
Yes	4614 (25)	3330 (23)	1284 (32)**
Central obesity	11 655 (64)	9113 (64)	2542 (63)
Alcohol use	10 051 (55)	7653 (54)	2398 (59)**
Physiological stress, *N* (%)			
Short sleep	5747 (31)	4230 (30)	1517 (38)**
Hypertension	5969 (33)	4466 (31)	1503 (37)**
Diabetes mellitus	1564 (9)	1118 (8)	446 (11)**
Dyslipidaemia	4157 (23)	3030 (21)	1127 (28)**
Psychosocial stress, *N* (%)			
Stress at work or home	4487 (25)	3405 (24)	1082 (27)**
CVD outcomes, *N* (%)			
CVD	2680 (15)	1963 (14)	717 (18)**

CVD, cardiovascular diseases; *N*, number; Q1, Q3, interquartile range; SD, standard deviation.

a Data only available in *N* = 3546.

** *P* < 0.01.

## RESULTS

The characteristics of the study population are presented in Table 1. Data on variables for all analyses were complete in 98% of participants (18 284 out of 18 746). Of these, 4311 were of Dutch origin (24%), 2704 of South-Asian Surinamese origin (15%), 3776 of African Surinamese origin (21%), 1925 of Ghanaian origin (11%), 2706 of Turkish origin (15%), and 2862 of Moroccan origin (16%). The mean age was 44 ± 12 years and 55% were female (9990 out of 18 284). Of these participants, 22% have to (or had to) work at irregular hours (4041 out of 18 284).

Table 2 presents the association between irregular working hours and prevalent CVD. There was a significant association between irregular working hours and CVD after adjustment for sociodemographic confounders (odds ratio [OR] 1.27, 95% CI 1.15–1.40). The association remained significant after additional adjustment for behavioural, physiological and psychosocial stress (OR 1.18, 95% CI 1.07–1.30). Psychosocial stress had the largest effect on the OR for prevalent CVD (NIE lower bound = 1.033), before physiological stress (NIE lower bound = 1.024) and behavioural stress (NIE lower bound = 1.016).

**Table 2.** Association between irregular working hours and prevalent cardiovascular diseases

	*N* = 18 284	
Irregular working hours	OR (95% CI)	NIE lower bound
Model 1: sociodemographics	1.27 (1.15, 1.40)**	reference
Model 1 + behavioural stress	1.25 (1.13, 1.38)**	1.016
Model 1 + physiological stress	1.24 (1.12, 1.36)**	1.024
Hypertension, diabetes mellitus, dyslipidaemia	1.26 (1.14, 1.39)**	1.008
Short sleep	1.25 (1.13, 1.38)**	1.016
Model 1 + psychosocial stress	1.23 (1.11, 1.35)**	1.033
Model 1 + all stress-factors	1.18 (1.07, 1.30)**	1.076

CI, confidence interval; NIE, natural indirect effect; OR, odds ratio.

Sociodemographics: age, sex, occupational level and ethnicity.

Behavioural stress: smoking, alcohol use, physical inactivity and central obesity.

Physiological stress: hypertension, diabetes mellitus, dyslipidaemia and short sleep.

Psychosocial stress: experienced stress at home or work.

** *P* < 0.01.

Table 3 presents the association between the frequency of irregular working hours and prevalent CVD. Information regarding the frequency of irregular working hours was available in 97% of participants 17 805 out of 18 284. The OR for CVD among individuals with ≤20 irregular working hours per week was significantly higher compared to individuals with no irregular working hours after adjustment for sociodemographic confounders, behavioural stress or physiological stress. The OR for CVD among individuals working >20 irregular working hours per week was significantly higher compared to individuals with no irregular working hours in all mediator-adjusted models. In individuals with >20 irregular working hours, perceived psychosocial stress had the largest indirect effect on the OR for prevalent CVD (NIE lower bound = 1.038). The characteristics of individuals with >20 irregular working hours versus ≤20 irregular working hours are shown in Supplementary Table 2 (available as Supplementary data at *Occupational Medicine* Online).

**Table 3.** Association between the frequency of irregular working hours and prevalent cardiovascular diseases

	None	≤20 h per week		>20 h per week	
	*N* = 14 259	*N* = 1587		*N* = 1959	
Irregular working hours		OR (95% CI)	NIE lower bound	OR (95% CI)	NIE lower bound
Model 1: sociodemographics	1 (ref)	1.21 (1.04, 1.40)*	reference	1.35 (1.19, 1.53)**	reference
Model 1 + behavioural stress	1 (ref)	1.21 (1.04, 1.40)*	1.000	1.32 (1.16, 1.49)**	1.023
Model 1 + physiological stress	1 (ref)	1.20 (1.02, 1.39)*	1.008	1.31 (1.15, 1.49)**	1.031
Hypertension, diabetes mellitus, dyslipidaemia	1 (ref)	1.21 (1.04, 1.41)*	1.000	1.33 (1.18, 1.52)**	1.015
Short sleep	1 (ref)	1.19 (1.02, 1.38)*	1.017	1.33 (1.17, 1.51)**	1.015
Model 1 + psychosocial stress	1 (ref)	1.16 (0.98, 1.35)	1.043	1.30 (1.15, 1.48)**	1.038
Model 1 + all stress factors	1 (ref)	1.15 (0.98, 1.34)	1.052	1.24 (1.09, 1.41)**	1.089

CI, confidence interval; NIE, natural indirect effect; OR, odds ratio.

Sociodemographics: age, sex, occupational level and ethnicity.

Behavioural stress: smoking, alcohol use, physical inactivity and central obesity.

Physiological stress: hypertension, diabetes mellitus, dyslipidaemia and short sleep.

Psychosocial stress: experienced stress at home or work.

** *P* < 0.01;

* *P* < 0.05 versus no irregular working hour.

Table 4 presents the sociodemographic and clinical characteristics of individuals with versus without irregular working hours, stratified by ethnicity. South-Asian Surinamese, African Suriname and Moroccans with irregular working hours had significantly higher rates of prevalent CVD compared to their counterparts with no irregular working hours (*P* < 0.01). The association between irregular working hours and prevalent CVD for each ethnicity is demonstrated in Table 5. There was a significant association between irregular working hours and CVD for South-Asian Surinamese (OR 1.47, 95% CI 1.18–1.82), African Surinamese (OR 1.29, 95% CI 1.06, 1.57), and Moroccans (OR 1.43, 95% CI 1.11–1.84) after adjustment for sociodemographic confounders. The association remained significant for South-Asian Surinamese (OR 1.34, 95% CI 1.08–1.68) and African Surinamese (OR 1.23, 95% CI 1.01–1.51) after adjustment for behavioural, physiological, and psychosocial stress. Physiological stress had the largest indirect effect on the OR for prevalent CVD in South-Asian Surinamese (NIE lower bound = 1.058), while psychosocial stress had the largest indirect effect on the OR in African Surinamese (NIE lower bound = 1.032) and Moroccans (NIE lower bound = 1.092). The effects of physiological stress on the OR for prevalent CVD in South-Asian Surinamese and African Surinamese were predominantly mediated by short sleep.

**Table 4.** Sociodemographic and clinical characteristics, stratified by ethnicity

	Dutch		South-Asian Surinamese		African Surinamese		Ghanaian		Turkish		Morcoccan	
Irregular working hours	No	Yes	No	Yes	No	Yes	No	Yes	No	Yes	No	Yes
*N*	3438	873	2043	661	2705	1071	1546	379	2212	494	2299	563
Irregular working hours												
Hours per week *N* (Q1, Q3)^a^	–	15 (6, 32)	–	30 (10, 40)	–	32 (16, 40)	–	32 (12, 40)	–	21 (8, 40)	–	24 (10, 38)
Sociodemographics												
Age, years(SD)	46 (13)	46 (14)	45 (12)	46 (12)	47 (12)	49 (11)**	44 (10)	45 (11)	39 (11)	39 (11)	38 (12)	41 (11)**
Female, *N* (%)	1919 (56)	294 (45)**	1221 (60)	235 (36)**	1738 (64)	563 (53)**	974 (63)	165 (44)**	1187 (54)	110 (22)**	1340 (58)	144 (26)**
Occupational level *N* (%)												
Missing	70 (2)	19 (2)	66 (3)	23 (4)	115 (4)	44 (4)	63 (4)	19 (5)	89 (4)	17 (3)	101 (4)	27 (5)
Elementary	58 (2)	16 (2)	222 (11)	60 (9)	179 (7)	74 (7)	968 (63)	198 (52)**	446 (20)	61 (12)**	411 (18)	73 (13)**
Lower	466 (14)	170 (20)**	624 (31)	277 (42)**	839 (31)	425 (40)**	331 (21)	108 (29)**	825 (37)	237 (48)**	687 (30)	255 (45)**
Medium	734 (21)	253 (29)**	607 (30)	213 (32)	897 (33)	385 (36)	125 (8)	40 (11)	508 (23)	127 (26)	644 (28)	148 (26)
Higher	1353 (39)	283 (32)**	405 (20)	70 (11)**	583 (22)	129 (12)**	45 (3)	9 (2)	249 (11)	37 (8)*	373 (16)	57 (10)**
Scientific	757 (22)	132 (15)**	119 (6)	18 (3)**	92 (3)	14 (1)**	14 (1)	5 (1)	95 (4)	15 (3)	83 (4)	3 (1)**
Behavioural stress, *N* (%)												
Physical inactivity	887 (25.8)	171 (20)**	971 (48)	278 (42)*	1096 (41)	339 (22)**	724 (47)	142 (38)**	1262 (57)	249 (50)**	1179 (51)	275 (49)
Smoking												
Never-smoker	1307 (38)	261 (30)**	1220 (60)	314 (48)**	1358 (50)	482 (45)**	1371 (89)	307 (81)**	972 (44)	157 (32)**	1647 (72)	289 (51)**
Ex-smoker	1351 (39)	337 (39)	274 (13)	111 (17)*	528 (20)	220 (21)	120 (8)	48 (13)**	444 (20)	106 (22)	321 (14)	125 (22)**
Yes	780 (23)	275 (32)**	549 (27)	236 (36)**	819 (30)	369 (35)*	55 (4)	24 (6)*	796 (36)	231 (47)**	331 (14)	149 (27)**
Central obesity	1863 (54)	482 (55)	1449 (71)	462 (70)	1761 (65)	714 (67)	1091 (71)	230 (61)**	1499 (68)	311 (63)*	1450 (63)	343 (61)
Alcohol use	3139 (91)	805 (92)	1178 (58)	382 (58)	1857 (69)	751 (70)	737 (48)	199 (48)	554 (25)	180 (36)**	188 (8)	81 (14)**
Physiological stress, *N* (%)												
Short sleep	518 (15)	165 (19)**	738 (36)	294 (45)**	1153 (43)	546 (51)**	620 (40)	194 (51)**	616 (28)	153 (31)	585 (26)	165 (29)
Hypertension	857 (25)	237 (27)	747 (37)	281 (43)**	1193 (44)	538 (50)**	808 (52)	205 (54)	507 (23)	126 (26)	354 (15)	116 (21)**
Diabetes mellitus	110 (3)	29 (3)	299 (15)	138 (21)**	267 (10)	146 (14)**	139 (9)	32 (8)	154 (7)	30 (6)	149 (7)	71 (13)**
Dyslipidaemia	629 (18)	198 (23)**	724 (35)	297 (45)**	473 (18)	225 (21)*	188 (12)	63 (17)*	641 (29)	176 (36)**	375 (16)	168 (30)**
Psychosocial stress, *N* (%)												
Stress at home or work	787 (23)	232 (27)*	573 (28)	193 (29)	545 (20)	229 (21)	221 (14)	65 (17)	657 (30)	171 (35)*	622 (27)	192 (34)**
CVD outcomes, *N* (%)												
CVD	268 (8)	78 (9)	378 (19)	179 (27)**	365 (14)	191 (18)**	193 (13)	58 (15)	433 (20)	99 (20)	326 (14)	112 (20)**

CVD, cardiovascular diseases; *N*, number; Q1, Q3 = interquartile range; SD, standard deviation.

a Data only available in Dutch: *N* = 761, South Asian Suriname: *N* = 568, African Suriname: *N* = 909, Ghanaian: *N* = 347, Turkish: *N* = 445, Moroccan: *N* = 516.

** *P* < 0.01;

* *P* < 0.05 versus no irregular working hours.

**Table 5.** Association between irregular working hours and prevalent cardiovascular diseases, stratified by ethnicity

	Dutch		South-Asian Surinamese		African Surinamese		Ghanaian		Turkish		Moroccan	
	*N* = 4311		*N* = 2704		*N* = 3776		*N* = 1925		*N* = 2706		*N* = 2862	
Irregular working hours	OR (95% CI)	NIE lower bound	OR (95% CI)	NIE lower bound	OR (95% CI)	NIE lower bound	OR (95% CI)	NIE lower bound	OR (95% CI)	NIE lower bound	OR (95% CI)	NIE lower bound
Model 1: sociodemographics	1.01 (0.77, 1.33)	reference	1.47 (1.18, 1.82)**	reference	1.29 (1.06, 1.57)*	reference	1.30 (0.94, 1.80)	reference	1.12 (0.87, 1.45)	reference	1.43 (1.11, 1.84)**	reference
Model 1 + behavioural stress	0.97 (0.74, 1.28)	1.041	1.42 (1.14, 1.77)**	1.035	1.29 (1.06, 1.57)*	1.000	1.26 (0.91, 1.75)	1.032	1.08 (0.84, 1.40)	1.037	1.40 (1.09, 1.80)**	1.021
Model 1 + physiological stress	1.00 (0.75, 1.32)	1.010	1.39 (1.11, 1.73)**	1.058	1.26 (1.03, 1.53)*	1.023	1.26 (0.91, 1.75)	1.032	1.12 (0.87, 1.45)	1.000	1.40 (1.09, 1.81)**	1.021
Hypertension, diabetes mellitus, dyslipidaemia	1.00 (0.76, 1.33)	1.010	1.43 (1.15, 1.78)**	1.028	1.29 (1.06, 1.57)*	1.000	1.27 (0.92, 1.77)	1.024	1.13 (0.88, 1.47)	0.991	1.41 (1.10, 1.82)**	1.014
Short sleep	1.00 (0.77, 1.32)	1.010	1.42 (1.15, 1.77)**	1.035	1.26 (1.03, 1.53)*	1.023	1.29 (0.93, 1.78)	1.008	1.11 (0.86, 1.43)	1.009	1.42 (1.11, 1.83)**	1.007
Model 1 + psychosocial stress	1.00 (0.76, 1.31)	1.010	1.45 (1.16, 1.80)**	1.014	1.25 (1.03, 1.53)*	1.032	1.25 (0.90, 1.74)	1.040	1.07 (0.82, 1.38)	1.047	1.31 (1.01, 1.69)*	1.092
Model 1 + all stress-factors	0.94 (0.71, 1.25)	1.074	1.34 (1.08, 1.68)**	1.097	1.23 (1.01, 1.51)*	1.049	1.18 (0.84, 1.65)	1.102	1.04 (0.80, 1.36)	1.077	1.27 (0.98, 1.64)	1.126

CI, confidence interval; NIE, natural indirect effect; OR, odds ratio.

Sociodemographics: age, sex, occupational level and ethnicity.

Behavioural stress: smoking, alcohol use, physical inactivity and central obesity.

Physiological stress: hypertension, diabetes mellitus, dyslipidaemia and short sleep.

Psychosocial stress: experienced stress at home or work.

** *P* < 0.01;

* *P* < 0.05.

## DISCUSSION

The present study demonstrates that (1) irregular working hours are associated with an increased prevalence of CVD in a multi-ethnic population; (2) the association between irregular working hours and CVD in this multi-ethnic population is mediated by behavioural, physiological and psychosocial stress, of which psychosocial stress has a dominant mediating effect; (3) We demonstrate greater ORs for prevalent CVD in individuals working/reporting >20 irregular working hours per week versus individuals with ≤20 or no irregular working hours; (4) We demonstrate ethnic disparities in the association between irregular working hours and CVD, as well as in the stress-related pathways that mediate these associations. Stratified by ethnicity, significant independent associations were found in South-Asian Surinamese, African Surinamese and Moroccans.

A strength of this study is that participants were selected entirely independent of occupational status, thus representing an unbiased sample of the Dutch workforce. Furthermore, we used individual-level information and the large numbers of individuals per ethnicity, allowing detailed stratification by ethnicity. There are also some limitations. We included individuals who documented that stress at work is ‘not applicable’ to their situation. A sensitivity analysis excluding these individuals did not significantly change the presented ORs. The cross-sectional design prevents to study the causality of associations between irregular working hours and CVD. However, it is unlikely that CVD is a risk factor for shift work. Data were partly gathered through self-reported questionnaires, which are sensitive to recall and response bias. Moreover, no specific timeframe for irregular working hours was given in the questionnaire. We cannot exclude the effects of residual confounding, and we did not correct for mediating factors such as inflammation or cardiac autonomic function. Moreover, we did not account for the duration individuals were exposed to irregular working hours or the type of shift work. This may have affected the outcomes, especially since there is a dose–response relation between irregular working hours and CVD [10, 21]. Also, the notorious healthy worker effect phenomenon may have affected the outcomes. In our analysis, we divided the mediating stress-related pathways as described by Knutsson and Boggild [22], which is probably a simplistic representation of the more complex and interrelated mediating pathways. Last of all, we used the difference method to determine the lower bound of the NIE as a measure of mediation. While formulas exist to estimate the mediated proportion of an effect for rare outcomes (<10% prevalence), it is very difficult to calculate this proportion for common outcomes such as prevalent CVD, as the OR is non-collapsible in covariate-adjusted models. Moreover, parametric mediation-analysis methods are not suited for complex relationships in uncontrolled observational cross-sectional settings [23. The NIE lower bound can be reliably estimated but must be treated with caution as the actual NIE size can be sometimes far from the lower bound.

The results from our study support previous evidence indicating that irregular working hours are associated with an increased risk of CVD [9, 10, 21]. Most of this evidence originates from cohorts with predominantly European descent or East Asian participants [9, 21]. It remains therefore unknown if the increased cardiovascular burden among shift workers can be extrapolated to multi-ethnic populations or to other ethnic minority groups. Results from a multi-ethnic Asian population in Singapore showed that night workers had an increased risk for prevalent CVD. However, this association attenuated after adjusting for ethnicity and low socioeconomic status [24]. Our findings demonstrate that the increased CVD risk in individuals with irregular work hours remains after adjusting for sociodemographic confounders and various mediating stress factors. The mechanisms by which shift work may increase the risk for cardiovascular diseases are multifactorial and complex. There is, however, general consensus that the factors that associate irregular working hours with CVD can roughly be divided into three different, but interrelated, mediating stress-related pathways. Namely, behavioural stress due to changes in lifestyle factors, physiological stress due to disruption of circadian rhythms, and psychosocial stress [22, 25]. Individuals with irregular working hours are more likely to have an adverse cardiovascular risk profile, including higher use of tobacco and alcohol, physical inactivity, sedentary lifestyle, and obesity [24, 26–30]. Circadian disturbance in shift workers is associated with adverse biological effects, including reduced sleep duration and sleeping disorders [31, 32], increased systemic inflammation [33, 34], disruption of glucose and lipid metabolism [35–37], and abnormal cardiac autonomic function [38]. Finally, irregular working hours have a negative impact on the work-life balance [39] and are associated with job-strain, effort-reward imbalance and lower job control [40]. We demonstrate that irregular working hours-­related psychosocial stress has a dominant influence on the risk for CVD compared to physiological and behavioural stress. Ho *et al*. demonstrated that the risk for CVD is predominately mediated through modifiable risk factors such as tobacco use, sleep duration and quality, adiposity and metabolic status. The cohort in the study by Ho *et al.* [41], however, involved predominantly whites (94%), which hampers direct comparison with our multi-ethnic study population.

In South-Asian and African Surinamese, the risk for prevalent CVD is predominately mediated by physiological stress, and in particular short sleep. In line with previous studies, we show that insufficient sleep durations are more prevalent in ethnic minority groups [42], and especially in South-Asian and African Surinamese with irregular working hours. Previous studies showed that short sleep contributes significantly to ethnic disparities in cardiometabolic health, independent from traditional CVD risk factors [43, 44]. We also demonstrate greater differences in traditional CVD risk factors, such as diabetes mellitus, dyslipidaemia and hypertension, between individuals from South-Asian Surinamese, African Surinamese and Moroccan origin with versus without irregular working hours, as compared to other ethnic groups. Lieu et al. [8] demonstrated that rotating shift work is independently associated with increased risk of hypertension in blacks, but not in whites. This inequity may be explained by racial differences in the circadian biology of blood pressure. A normal circadian rhythm includes a nocturnal dip in blood pressure. There is evidence that the nocturnal dipping in blood pressure is blunted in African Americans and that rotating shift work further blunts the dip in blood pressure [45]. Future studies should examine if adequate sleep or more favourable work schedules in ethnic/racial groups mitigate the increased risk for adverse cardiometabolic health. Our study also suggests a direct effect of irregular working hours on prevalent CVD in South-Asian Surinamese and African Surinamese, indicating that additional factors mediate the relationship between irregular working hours and CVD in these ethnic groups. Systemic inflammation and cardiac autonomic dysfunction are both associated with circadian misalignment, and may mediate the risk for CVD in individuals with irregular working hours [34, 38, 46]. We show significantly higher levels of psychosocial stress in Moroccans working irregular working hours. Psychosocial stress is an independent risk factor for CVD, which may explain the profound mediating effect among Moroccans [47]. Our study documented that a higher frequency of irregular working hours is associated with an increased risk for CVD, which is in line with previous observations that the cardiovascular burden increases with increasing exposure to irregular working hours [10, 48]. This may also explain the absence of an association in Dutch individuals, as the Dutch reported a relatively low number of irregular working hours per week.

Our results may have profound implications for our policies to prevent CVD in shift workers. Ethnicity-tailored preventive strategies may have the potential to improve the prevention of CVD in shift workers. Future studies should therefore explore if preventive strategies that take ethnic origin into consideration, such as ethnicity-tailored shift worker health surveillance programs, result in improved prevention of CVD among individuals with irregular working hours. In conclusion, working irregular hours is associated with an increased prevalence of CVD in a multi-ethnic population living in Amsterdam, The Netherlands. We show ethnic disparities in the associations between irregular working hours and CVD, as well as, in the stress pathways that mediate these associations.

## Supplementary Material

kqaf069_Supplementary_Data

## Data Availability

The HELIUS data are owned by the Amsterdam University Medical Centers, located at AMC in Amsterdam, The Netherlands. Any researcher can request the data by submitting a proposal to the HELIUS Executive Board as outlined at http://www.heliusstudy.nl/en/researchers/collaboration, by email: heliuscoordinator@amsterdamumc.nl. The HELIUS Executive Board will check proposals for compatibility with the general objectives, ethical approvals and informed consent forms of the HELIUS study. There are no other restrictions on obtaining the data, and all data requests will be processed in the same manner.
