# Supplementary material for: The association between irregular working hours and cardiovascular diseases in a multi-ethnic population: the HELIUS study
Source: Occup Med (Lond). 2025 Jul 11;75(7):442–50. doi: 10.1093/occmed/kqaf069 (PMC12513472; doi:10.1093/occmed/kqaf069)
Supplement: kqaf069_Supplementary_Data [file kqaf069_supplementary_data.zip › 24-OP-249.R3a supplementary tables FINAL.doc]

| **Supplementary table 1:** **Interaction between irregular working hours and ethnicity on cardiovascular diseases** | | | | | |
| --- | --- | --- | --- | --- | --- |
|  | Sociodemographic condounders (model 1) | Model1 + Behavioural stress | Model 1 + Physiological stress | Model 1 +  Psychosocial stress | Model 1 + all stressfactors |
| Ethnic group | OR (95%-CI) | OR (95%-CI) | OR (95%-CI) | OR (95%-CI) | OR (95%-CI) |
| Dutch | 1.00 | 1.00 | 1.00 | 1.00 | 1.00 |
| South-Asian Surinamese | 1.41 (1.01, 1.98)* | 1.40 (1.00, 1.96) | 1.38 (0.98, 1.94) | 1.44 (1.03, 2.02)* | 1.39 (0.99, 1.96) |
| African Surinamese | 1.15 (0.83, 1.60) | 1.18 (0.85, 1.65) | 1.16 (0.83, 1.62) | 1.16 (0.84, 1.62) | 1.20 (0.86, 1.67) |
| Ghanaian | 1.14 (0.75, 1.73) | 1.18 (0.78, 1.80) | 1.14 (0.75, 1.73) | 1.14 (0.75, 1.73) | 1.16 (0.76, 1.77) |
| Turkish | 0.94 (0.65, 1.35) | 0.94 (0.65, 1.36) | 0.95 (0.66, 1.38) | 0.92 (0.64, 1.32) | 0.94 (0.65, 1.36) |
| Moroccan | 1.25 (0.87, 1.78) | 1.20 (0.84, 1.73) | 1.24 (0.87, 1.77) | 1.20 (0.83, 1.72) | 1.16 (0.80, 1.67) |
| OR=Odds ratio, CI=Confidence interval | | | | | |
| **p<0.01; *p<0.05 | | | | | |
| Sociodemographics: age, sex, occupational level, ethnicity | | | | | |
| Behavioral stress: smoking, alcohol use, physical inactivity, central obesity | | | | | |
| Physiological stress: hypertension, diabetes mellitus, dyslipidemia, short sleep | | | | | |
| Psychosocial stress: experienced stress at home or work | | | | | |

| **Supplementary table 2: Sociodemographic and clinical characteristics for individual with >20 versus ≤20 irregular working hours** | | | | |
| --- | --- | --- | --- | --- |
|  | | | ≤20 hours per week | >20 hours per week |
|  |  |  |  |  |
| N | | | 1,587 | 1,959 |
| **Irregular working hours** | | |  |  |
|  | Hours per week N (Q1, Q3)ᵻ | | 8 (5, 15) | 38 (32, 40) |
| **Ethnicity, N(%)** | | |  |  |
|  | Dutch | | 477 (30.1) | 284 (14.5) |
|  | South-Asian Surinamese | | 231 (14.6) | 337 (17.2) |
|  | African Surinamese | | 309 (19.5) | 600 (30.6) |
|  | Ghanaian | | 116 (7.3) | 231 (11.8) |
|  | Turkish | | 222 (14.0) | 223 (11.4) |
|  | Moroccan | | 232 (14.6) | 284 (14.5) |
| **Sociodemographics** | | |  |  |
|  | Age, years (SD) | | 42 (13) | 47 (11) |
|  | female, N (%) | | 633 (39.9) | 725 (37.0) |
|  | Occupational level, N (%) | |  |  |
|  |  | missing | 50 (3.2) | 74 (3.8) |
|  |  | elementary | 153 (9.6) | 273 (13.9) |
|  |  | lower | 512 (32.3) | 781 (39.9) |
|  |  | medium | 433 (27.3) | 586 (29.9) |
|  |  | higher | 305 (19.2) | 214 (10.9) |
|  |  | scientific | 134 (8.4) | 31 (1.6) |
| **Behavioral stress, N (%)** | | |  |  |
|  | Physical inactivity | | 524 (33.0) | 757 (38.6) |
|  | Smoking | |  |  |
|  |  | never-smoker | 720 (45.4) | 871 (44.5) |
|  |  | ex-smoker | 373 (23.5) | 450 (23.0) |
|  |  | yes | 494 (31.1) | 638 (32.6) |
|  | Central obesity | | 883 (55.6) | 1,311 (66.9) |
|  | Alcohol use | | 997 (62.8) | 1,126 (57.5) |
| **Physiological stress, N (%)** | | |  |  |
|  | Short sleep | | 517 (32.6) | 805 (41.1) |
|  | Hypertension | | 451 (28.4) | 846 (43.2) |
|  | Diabetes Mellitus | | 123 (7.8) | 252 (12.9) |
|  | Dyslipidemia | | 370 (23.3) | 607 (31.0) |
| **Psychosocial stress, N (%)** | | |  |  |
|  | Stress at work or home | | 449 (28.3) | 510 (26.0) |
| **CVD outcomes, N (%)** | | |  |  |
|  | CVD | | 240 (15.1) | 392 (20.0) |
| N=number, Q1, Q3=interquartile range, SD=standard deviatio,CVD=cardiovascular diseases | | |  |  |
| **p<0.01; *p<0.05 | | | | |

| **Supplementary table 3: Sociodemographic and clincial characteristics for individual with >20 versus ≤20 irregular working hours, stratified by ethnicity** | | | | | | | | | | | | | | | | | | |  |
| --- | --- | --- | --- | --- | --- | --- | --- | --- | --- | --- | --- | --- | --- | --- | --- | --- | --- | --- | --- |
|  |  |  | **Dutch** | |  | **South-Asian Surinamese** | |  | **African Surinamese** | |  | **Ghanaian** | |  | **Turkish** | |  | **Morcoccan** | |
|  |  |  | ≤20 hours per week | >20 hours per week |  | ≤20 hours per week | >20 hours per week |  | ≤20 hours per week | >20 hours per week |  | ≤20 hours per week | >20 hours per week |  | ≤20 hours per week | >20 hours per week |  | ≤20 hours per week | >20 hours per week |
| N | | | 477 | 284 |  | 231 | 337 |  | 309 | 600 |  | 116 | 231 |  | 222 | 223 |  | 232 | 284 |
| **Irregular working hours** | | |  |  |  |  |  |  |  |  |  |  |  |  |  |  |  |  |  |
|  | Hours per week N (Q1, Q3) | | 8 (4, 12) | 36 (32, 40) |  | 8 (6, 16) | 38 (32, 40) |  | 9 (6, 16) | 36 (32, 40) |  | 8 (8, 13) | 38 (28, 40) |  | 8 (5, 15) | 36 (32, 40) |  | 8 (7, 16) | 36 (32, 40) |
| **Sociodemographics** | | |  |  |  |  |  |  |  |  |  |  |  |  |  |  |  |  |  |
|  | Age, years(SD) | | 42 (13) | 51 (13) |  | 42 (12) | 48 (11) |  | 47 (13) | 50 (10) |  | 40 (13) | 47 (9) |  | 37 (12) | 41 (11) |  | 39 (12) | 42 (11) |
|  | female, N(%) | | 220 (46.1) | 118 (41.6) |  | 82 (35.5) | 106 (31.5) |  | 154 (49.8) | 303 (50.5) |  | 63 (54.3) | 90 (39.0) |  | 55 (24.8) | 43 (19.3) |  | 59 (25.4) | 65 (22.9) |
|  | Occupational level, N(%) | |  |  |  |  |  |  |  |  |  |  |  |  |  |  |  |  |  |
|  |  | Missing | 8 (1.7) | 8 (2.8) |  | 5 (2.2) | 13 (3.9) |  | 11 (3.6) | 23 (3.8) |  | 7 (6.0) | 9 (3.9) |  | 8 (3.6) | 5 (2.2) |  | 11 (4.7) | 16 (5.6) |
|  |  | elementary | 8 (1.7) | 6 (2.1) |  | 15 (6.5) | 34 (10.1) |  | 29 (9.4) | 33 (5.5) |  | 50 (43.1) | 130 (56.3) |  | 26 (11.7) | 27 (12.1) |  | 25 (10.8) | 43 (15.1) |
|  |  | lower | 73 (15.3) | 77 (27.1) |  | 89 (38.5) | 149 (44.2) |  | 112 (36.3) | 247 (41.2) |  | 41 (35.3) | 60 (26.0) |  | 95 (42.8) | 116 (52.0) |  | 102 (44.0) | 132 (46.5) |
|  |  | medium | 116 (24.3) | 93 (32.8) |  | 71 (30.7) | 114 (33.8) |  | 108 (35.0) | 223 (37.2) |  | 13 (11.2) | 25 (10.8) |  | 60 (28.0) | 61 (27.0) |  | 65 (28.0) | 70 (24.7) |
|  |  | higher | 171 (35.9) | 80 (28.2) |  | 39 (16.9) | 25 (7.4) |  | 44 (14.2) | 67 (11.2) |  | 2 (1.7) | 7 (3.0) |  | 22 (9.9) | 13 (5.8) |  | 27 (11.6) | 22 (7.8) |
|  |  | scientific | 101 (21.2) | 20 (7.0) |  | 12 (5.2) | 2 (0.6) |  | 5 (1.6) | 7 (1.2) |  | 3 (2.6) | - |  | 11 (5.0) | 1 (0.5) |  | 2 (0.9) | 1 (0.4) |
| **Behavioral stress, N (%)** | | |  |  |  |  |  |  |  |  |  |  |  |  |  |  |  |  |  |
|  | Physical inactivity | | 99 (20.8) | 51 (18.0) |  | 92 (39.8) | 147 (43.6) |  | 88 (28.5) | 202 (33.7) |  | 38 (32.8) | 89 (38.5) |  | 100 (45.1) | 121 (54.3) |  | 107 (46.2) | 147 (51.8) |
|  | Smoking | |  |  |  |  |  |  |  |  |  |  |  |  |  |  |  |  |  |
|  |  | never-smoker | 150 (31.5) | 81 (28.5) |  | 116 (50.2) | 151 (44.8) |  | 152 (49.2) | 253 (42.2) |  | 98 (84.8) | 182 (78.8) |  | 81 (36.5) | 63 (28.3) |  | 123 (53.0) | 141 (49.9) |
|  |  | ex-smoker | 172 (36.1) | 119 (41.9) |  | 34 (14.7) | 59 (17.5) |  | 57 (18.5) | 135 (22.5) |  | 15 (12.9) | 30 (13.0) |  | 44 (19.8) | 46 (20.6) |  | 51 (22.0) | 61 (21.5) |
|  |  | yes | 155 (32.5) | 84 (29.6) |  | 81 (35.1) | 127 (37.7) |  | 100 (32.4) | 212 (35.3) |  | 3 (2.6) | 19 (8.2) |  | 97 (43.7) | 114 (51.1) |  | 58 (25.0) | 82 (28.9) |
|  | Central obesity | | 221 (46.3) | 189 (66.6) |  | 145 (62.8) | 240 (71.2) |  | 192 (62.1) | 406 (67.7) |  | 66 (56.9) | 145 (62.8) |  | 126 (56.8) | 153 (68.6) |  | 133 (57.3) | 178 (62.7) |
|  | Alcohol use | | 451 (94.6) | 254 (89.6) |  | 142 (61.5) | 196 (58.2) |  | 227 (73.5) | 428 (71.3) |  | 58 (50.0) | 126 (54.6) |  | 81 (36.5) | 84 (37.7) |  | 38 (16.4) | 38 (13.4) |
| **Physiological stress, N (%)** | | |  |  |  |  |  |  |  |  |  |  |  |  |  |  |  |  |  |
|  | Short sleep | | 81 (17.0) | 58 (20.4) |  | 91 (39.4) | 159 (47.2) |  | 159 (51.5) | 304 (50.7) |  | 55 (47.4) | 130 (56.3) |  | 59 (26.6) | 77 (34.5) |  | 72 (31.0) | 77 (27.1) |
|  | Hypertension | | 98 (20.6) | 108 (38.0) |  | 77 (33.3) | 158 (46.9) |  | 134 (43.4) | 324 (54.0) |  | 46 (39.7) | 139 (60.2) |  | 48 (21.6) | 59 (26.5) |  | 48 (20.7) | 58 (20.4) |
|  | Diabetes mellitus | | 7 (1.5) | 17 (6.0) |  | 33 (14.3) | 78 (23.2) |  | 35 (11.3) | 89 (14.8) |  | 9 (7.8) | 21 (9.1) |  | 12 (5.4) | 10 (4.5) |  | 27 (11.6) | 37 (13.0) |
|  | Dyslipidemia | | 84 (17.6) | 89 (31.3) |  | 84 (36.4) | 162 (48.1) |  | 54 (17.5) | 141 (23.5) |  | 17 (14.7) | 39 (16.9) |  | 69 (31.1) | 87 (39.0) |  | 62 (26.7) | 89 (31.3) |
| **Psychosocial stress, N (%)** | | |  |  |  |  |  |  |  |  |  |  |  |  |  |  |  |  |  |
|  | Stress at home or work | | 130 (27.3) | 76 (26.8) |  | 74 (32.0) | 95 (28.2) |  | 72 (23.3) | 120 (20.0) |  | 24 (20.7) | 34 (14.7) |  | 70 (31.5) | 88 (39.5) |  | 79 (34.1) | 97 (34.2) |
| **CVD outcomes, N (%)** | | |  |  |  |  |  |  |  |  |  |  |  |  |  |  |  |  |  |
|  | CVD | | 41 (8.6) | 31 (10.9) |  | 51 (22.1) | 101 (30.0) |  | 49 (15.9) | 113 (18.8) |  | 16 (13.8) | 37 (16.0) |  | 37 (16.7) | 50 (22.4) |  | 46 (19.8) | 60 (21.1) |
| Q1, Q3=interquartile range, N=number, SD=standard deviation, CVD=cardiovascular diseases | | | | | | | | | | | | | | | | | | |  |
| **p<0.01; *p<0.05 versus no irregular working hours | | | | | | | | | | | | | | | | | | |  |

| **Supplementary table 4: Association between frequency of irregular working hours and prevalent cardiovascular diseases, stratified per ethnicity** | | | | | | | | | | | | | | | | | | | | |  |
| --- | --- | --- | --- | --- | --- | --- | --- | --- | --- | --- | --- | --- | --- | --- | --- | --- | --- | --- | --- | --- | --- |
|  | **Dutch (N=4,205)** | | | | | |  | | **South-Asian Surinamese (N=2,614)** | | | |  | | **African Surinamese (N=3,617)** | | | | |  | |
| Irregular working hours | | | | ≤20 hours per week | | >20 hours per week | |  | | ≤20 hours per week | | >20 hours per week | |  | | ≤20 hours per week | | >20 hours per week | | | |
|  |  |  |  | N=477 | | N=284 | |  | | N=231 | | N=337 | |  | | N=309 | | N=600 | | | |
|  | | | | OR (95%-CI) | NIE lower bound | OR (95%-CI) | NIE lower bound |  | | OR (95%-CI) | NIE lower bound | OR (95%-CI) | NIE lower bound |  | | OR (95%-CI) | NIE lower bound | OR (95%-CI) | NIE lower bound | | |
|  | Sociodemographics | | | 1.23 (0.86, 1.75) | reference | 0.97 (0.64, 1.46) | reference |  | | 1.28 (0.91, 1.79) | reference | 1.54 (1.18, 2.03)** | reference |  | | 1.20 (0.86, 1.67) | reference | 1.38 (1.08, 1.75)** | reference | | |
|  | + Behavioral stress | | | 1.19 (0.83, 1.71) | 1.034 | 0.92 (0.61, 1.39) | 1.054 |  | | 1.27 (0.90, 1.79) | 1.008 | 1.49 (1.13, 1.96)** | 1.034 |  | | 1.23 (0.88, 1.71) | 0.976 | 1.36 (1.07, 1.73)* | 1.015 | | |
|  | + Physiological stress | | | 1.23(0.85, 1.76) | 1.000 | 0.91 (0.60, 1.39) | 1.066 |  | | 1.26 (0.89, 1.77) | 1.016 | 1.47 (1.11, 1.94)** | 1.048 |  | | 1.18 (0.84, 1.65) | 1.017 | 1.32 (1.03, 1.67)* | 1.045 | | |
|  |  | | Hypertension, diabetes mellitus, dyslipidemia | 1.24 (0.86, 1.78) | 0.992 | 0.91 (0.60, 1.39) | 1.066 |  | | 1.29 (0.91, 1.82) | 0.992 | 1.52 (1.16, 2.01)** | 1.013 |  | | 1.22 (0.87, 1.70) | 0.984 | 1.34 (1.06, 1.71)* | 1.030 | | |
|  |  | | Short sleep | 1.22 (0.85, 1.73) | 1.008 | 0.97 (0.64, 1.46) | 1.000 |  | | 1.25 (0.88, 1.76) | 1.024 | 1.49 (1.13, 1.95)** | 1.034 |  | | 1.16 (0.84, 1.62) | 1.034 | 1.35 (1.06, 1.71)* | 1.022 | | |
|  | + Psychosocial stress | | | 1.21 (0.85, 1.73) | 1.017 | 0.95 (0.63, 1.43) | 1.021 |  | | 1.24 (0.88, 1.75) | 1.032 | 1.52(1.16, 2.00)** | 1.013 |  | | 1.14 (0.82, 1.60) | 1.053 | 1.35 (1.06, 1.71)* | 1.022 | | |
|  | + All stressfactors | | | 1.16 (0.81, 1.68) | 1.060 | 0.87 (0.57, 1.32) | 1.115 |  | | 1.23 (0.87, 1.75) | 1.041 | 1.41 (1.06, 1.87)* | 1.092 |  | | 1.17 (0.84, 1.65) | 1.026 | 1.29 (1.01, 1.65)* | 1.070 | | |
|  | | | |  |  |  |  |  | |  |  |  |  |  | |  |  |  |  | | |
|  | | | | **Ghanaian (N=1,894)** | | | |  | | **Turkish (N=2,658)** | | | |  | | **Moroccan (N=2,817)** | | | | | |
| Irregular working hours | | | | ≤20 hours per week | | >20 hours per week | |  | | ≤20 hours per week | | >20 hours per week | |  | | ≤20 hours per week | | >20 hours per week | | | |
|  |  |  |  | N=116 | | N=231 | |  | | N=222 | | N=223 | |  | | N=232 | | N=284 | | | |
|  | | | | OR (95%-CI) | NIE lower bound | OR (95%-CI) | NIE lower bound |  | | OR (95%-CI) | NIE lower bound | OR (95%-CI) | NIE lower bound |  | | OR (95%-CI) | NIE lower bound | OR (95%-CI) | NIE lower bound | | |
|  | Sociodemographics | | | 1.23 (0.70, 2.14) | reference | 1.34 (0.90, 1.98) | reference |  | | 0.95 (0.65, 1.38) | reference | 1.24 (0.88, 1.75 | reference |  | | 1.48 (1.04, 2.11)* | reference | 1.53 (1.11, 2.10)* | reference | | |
|  | + Behavioral stress | | | 1.18 (0.68, 2.07) | 1.042 | 1.30 (0.87, 1.93) | 1.031 |  | | 0.95 (0.65, 1.38) | 1.000 | 1.17 (0.82, 1.65) | 1.060 |  | | 1.46 (1.03, 2.09)* | 1.014 | 1.48 (1.08, 2.05)* | 1.034 | | |
|  | + Physiological stress | | | 1.19 (0.68, 2.08) | 1.034 | 1.30 (0.88, 1.94) | 1.031 |  | | 0.97 (0.66, 1.42) | 0.979 | 1.23 (0.87, 1.74) | 1.008 |  | | 1.42 (1.00, 2.03) | 1.042 | 1.53 (1.11, 2.12)* | 1.000 | | |
|  |  | | Hypertension, diabetes mellitus, dyslipidemia | 1.20 (0.69, 2.11) | 1.025 | 1.32 (0.89, 1.96) | 1.015 |  | | 0.96 (0.66, 1.40) | 0.990 | 1.26 (0.89, 1.78) | 0.984 |  | | 1.45 (1.02, 2.07)* | 1.021 | 1.52 (1.10, 2.10)* | 1.007 | | |
|  |  | | Short sleep | 1.22 (0.69, 2.12) | 1.008 | 1.32 (0.89, 1.95) | 1.015 |  | | 0.95 (0.65, 1.39) | 1.000 | 1.21 (0.86, 1.71) | 1.025 |  | | 1.45 (1.02, 2.07)* | 1.021 | 1.54 (1.12, 2.13)* | 0.994 | | |
|  | + Psychosocial stress | | | 1.14 (0.65, 2.02) | 1.079 | 1.27 (0.85, 1.91) | 1.055 |  | | 0.92 (0.63, 1.35) | 1.033 | 1.14 (0.81, 1.61) | 1.088 |  | | 1.35 (0.94, 1.94) | 1.096 | 1.40 (1.01, 1.93)* | 1.093 | | |
|  | + All stressfactors | | | 1.06 (0.60, 1.88) | 1.160 | 1.25 (0.84, 1.88) | 1.072 |  | | 0.95 (0.65, 1.39) | 1.000 | 1.08 (0.76, 1.54) | 1.148 |  | | 1.29 (0.90, 1.86) | 1.147 | 1.37 (0.99, 1.86) | 1.117 | | |
|  | | OR=Odds ratio, CI=Confidence interval, NIE=natural indirect effect | | | | | | | | | | | | | | | | | | |  |
|  | | **p<0.01; *p<0.05 versus no irregular working hours | | | | | | | | | | | | | | | | | | |  |
|  | | Sociodemographics: age, sex, occupational level | | | | | | | | | | | | | | | | | | |  |
|  | | Behavioral stress: smoking, alcohol use, physical inactivity, central obesity | | | | | | | | | | | | | | | | | | |  |
|  | | Physiological stress: hypertension, diabetes mellitus, dyslipidemia, short sleep | | | | | | | | | | | | | | | | | | |  |
|  | | Psychosocial stress: experienced stress at home or work | | | | | | | | | | | | | | | | | | |  |
